# Profound Re-Organization of Cell Surface Proteome in Equine Retinal Pigment Epithelial Cells in Response to *In Vitro* Culturing

**DOI:** 10.3390/ijms131114053

**Published:** 2012-10-31

**Authors:** Christoph M. Szober, Stefanie M. Hauck, Kerstin N. Euler, Kristina J. H. Fröhlich, Claudia Alge-Priglinger, Marius Ueffing, Cornelia A. Deeg

**Affiliations:** 1 Institute of Animal Physiology, Department of Veterinary Sciences, Ludwig-Maximilians-University Munich, D-80539 Munich, Germany; E-Mails: christoph.szober@tiph.vetmed.uni-muenchen.de (C.M.S.); kerstin.euler@tiph.vetmed.uni-muenchen.de (K.N.E.); k.froehlich@tiph.vetmed.uni-muenchen.de (K.J.H.F.); 2 Research Unit Protein Science, Helmholtz Center Munich, German Research Center for Environmental Health, D-85764 Neuherberg, Germany; E-Mails: hauck@helmholtz-muenchen.de (S.M.H.); marius.ueffing@helmholtz-muenchen.de (M.U.); 3 Department of Ophthalmology, Ludwig-Maximilians-University, Mathildenstrasse 8, D-80336 Munich, Germany; E-Mail: claudia.alge-priglinger@med.uni-muenchen.de; 4 Centre of Ophthalmology, Institute for Ophthalmic Research, University of Tübingen, Röntgenweg 11, D-72076 Tübingen, Germany

**Keywords:** equine recurrent uveitis, membrane protein, outer blood-retinal barrier, retinal pigment epithelium cells, cell line, proteomics

## Abstract

The purpose of this study was to characterize the cell surface proteome of native compared to cultured equine retinal pigment epithelium (RPE) cells. The RPE plays an essential role in visual function and represents the outer blood-retinal barrier. We are investigating immunopathomechanisms of equine recurrent uveitis, an autoimmune inflammatory disease in horses leading to breakdown of the outer blood-retinal barrier and influx of autoreactive T-cells into affected horses’ vitrei. Cell surface proteins of native and cultured RPE cells from eye-healthy horses were captured by biotinylation, analyzed by high resolution mass spectrometry coupled to liquid chromatography (LC MS/MS), and the most interesting candidates were validated by PCR, immunoblotting and immunocytochemistry. A total of 112 proteins were identified, of which 84% were cell surface membrane proteins. Twenty-three of these proteins were concurrently expressed by both cell states, 28 proteins exclusively by native RPE cells. Among the latter were two RPE markers with highly specialized RPE functions: cellular retinaldehyde-binding protein (CRALBP) and retinal pigment epithelium-specific protein 65kDa (RPE65). Furthermore, 61 proteins were only expressed by cultured RPE cells and absent in native cells. As we believe that initiating events, leading to the breakdown of the outer blood-retinal barrier, take place at the cell surface of RPE cells as a particularly exposed barrier structure, this differential characterization of cell surface proteomes of native and cultured equine RPE cells is a prerequisite for future studies.

## 1. Introduction

Protein expression analysis with mass spectrometry has become an important tool to understand participating proteins in physiological networks and on cellular levels [1]. The proteome is the set of expressed proteins in a given type of cell at a given time under defined conditions [2]. Analysis technologies continually improved in the field of proteomics in the last years, especially regarding sensitivity of mass spectrometric analysis [3]. This enables increasingly better investigations of complex and widely dynamic protein concentrations [3]. Two-dimensional polyacrylamide gel electrophoresis (2D-PAGE) allowed resolution of thousands of proteins [4], but it also has major disadvantages, mainly inadequate resolution of cell surface membrane proteins. Recently, several different approaches were applied to enhance cell surface membrane protein identification in tissues through variations in sample preparation [5,6]. Further, mass spectrometry is rapidly advancing, and liquid chromatography coupled to tandem mass spectrometry (LC-MS/MS) has emerged as a highly precise and sensitive high-throughput technique for protein identification and characterization and facilitates discovery of proteins [3,6].

We are interested in immunopathomechanisms of equine recurrent uveitis (ERU), a spontaneous, organ-specific autoimmune disease characterized through remitting-relapsing attacks of leukocytes to the retina [1]. Earlier examinations of immune and target tissue proteomes revealed several interesting findings in this model disease for human autoimmune uveitis, e.g., identification of a novel autoantigen with importance for ERU and correspondent human disease [7,8]. Another surprising finding was the involvement of regulated granulocytes in intraocular inflammation of this T-cell mediated disease [4]. Moreover, differential proteome analyses of changes in targeted retina revealed a decisive role for retinal Mueller glial cells in pathogenesis [1,9,10]. A key event of uveitis is migration of immune cells through the blood-retinal barrier, because the inner eye is physiologically devoid of leukocytes. There are two different structures of blood-retinal barriers in the eye. Inner blood-retinal barrier is formed by two beds of capillary endothelia [11]. Outer blood-retinal barrier lies on the outer surface of the photoreceptor layer and closely resembles the ventricular portion of the blood-brain barrier [11]. Horses possess a widely avascular retina [12], and activated cells pass outer blood-retinal barrier in ERU [13]. Outer blood-retinal barrier is formed and maintained by retinal pigment epithelium (RPE) cells [11]. RPE cells use different mechanisms to establish important barrier function, including membrane pumps, transporters and different channels [11]. Initiating events of blood-retinal barrier breakdown are expected to take place at the cell surface as a particularly exposed barrier structure. So far, there is only very limited knowledge about the cell surface membrane proteome of retinal pigment epithelium cells, and therefore, the goal of this study was the identification of physiological RPE cell surface protein expression. Since studies for RPE cell functions are mainly performed with RPE cell lines [14], we additionally examined changes in RPE cell surface protein expression induced through cultivation of cells to evaluate modifications of RPE cells after passaging.

## 2. Results

### 2.1. Cell Surface Protein Expression of Physiological RPE Cells

**Figure 1:**
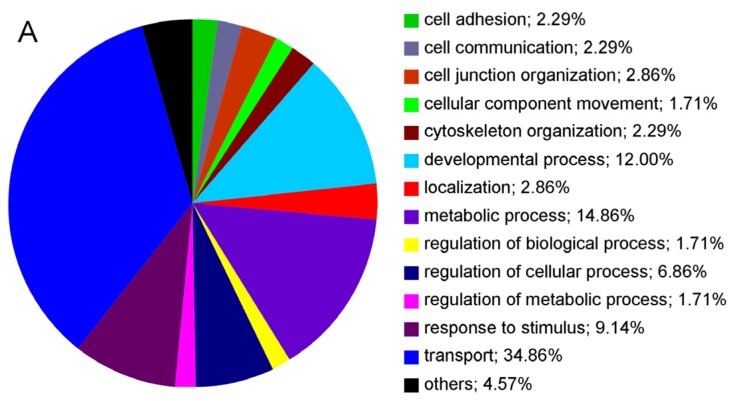

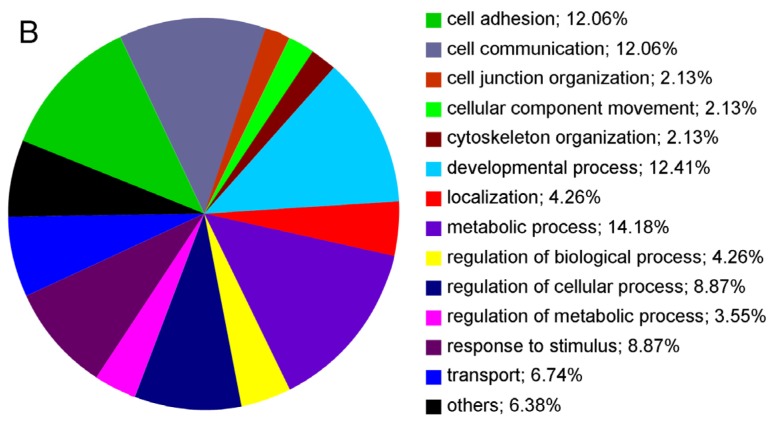
Classification of RPE cell surface proteins based on GO for biological process of native RPE cell proteins (**A**) and passage-4 RPE cell proteins (**B**). Numbers in percentages (%) correspond to the number of GO terms assigned for respective preparation. GO terms defined as others include those with an occurrence of <1.1%.

**Figure 2:**
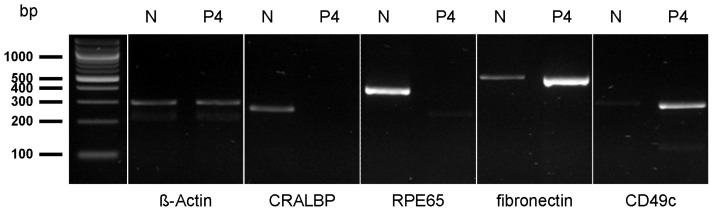
Gel electrophoresis of PCR products of native (N) and passage-4 (P4) equine RPE cell cDNA on a 2% agarose gel. Beta-actin is equally expressed by both cell states. Native RPE cells express CRALBP and RPE65, whereas no signal could be detected in passage-4 RPE. Fibronectin and CD49c are expressed distinctly by passage-4 RPE cells, while only a faint signal can be seen in native cells.

**Figure 3:**
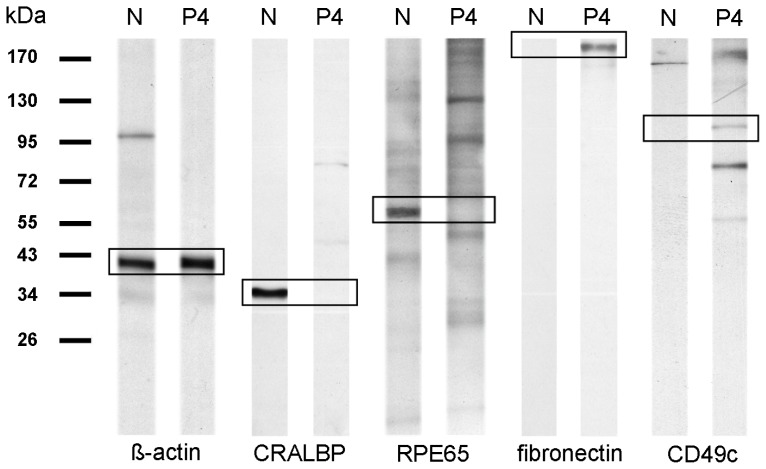
Western blot of a 10% 1D SDS-gel with lysates from native and cultured equine RPE passage-4 cells. N = native; P4 = passage-4. 5 μg protein of each lysate were loaded per lane. Boxes indicate the expected region for a signal regarding the molecular size of the respective protein.

A total of 112 proteins were identified with two or more peptides on the cell surfaces of RPE cells derived from healthy eyes (Table 1). Of these identified proteins, 84% were membrane proteins according to GO terminology (Table 1, cellular localization and Figure 1). Major differences were seen in proteins with GO for transport dominated in native cells (Figure 1A, bright blue segment) whereas proteins assigned with cell adhesion (Figure 1, dark green) and cell communication (Figure 1, grey) were upregulated in P4 cells (Figure 1B) in comparison to native RPE cells (Figure 1A). The relation between the last two GO terms, cell adhesion and cell communication, to all GO for biological process remained the same (Figure 1A,B, dark green and grey segments). In detail, 23 proteins were equally detectable in both examined states, native and passage-4 cells (20.5% of all 112 identified proteins), (Table 1, proteins 1–23). Beta-actin was among these unchanged proteins under both conditions, as seen in PCR and Western blot analysis, indicating equal protein loading for mass spectrometry (Table 1, protein 3; Figures 2 and 3). Besides some keratins (Table 1; proteins 7–12), an integrin (Table 1; protein 4) and six ion channel proteins (Table 1; proteins 15–17, 21–23) were stably expressed in native and cultured RPE cells.

### 2.2. Cell Surface Proteomes of Native and Cultured RPE Cells Differ Considerably

**Figure 4:**
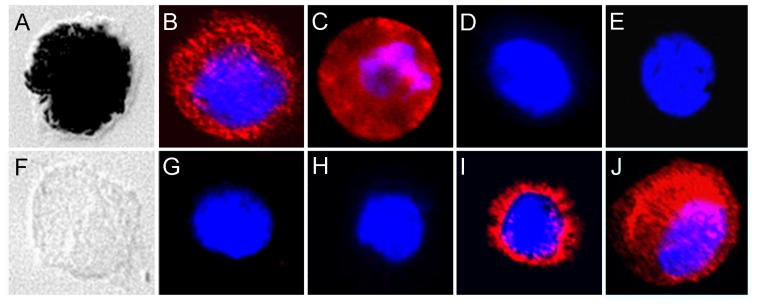
Immunocytochemistry of equine RPE cells. Upper panel (**A**–**E**) shows native equine RPE cells. Lower panel (**F**–**J**) shows cultured equine RPE cells of passage-4. Native cells show positive immunoreactivity for CRALBP (**B**) and RPE65 (**C**). Native cells show no immunoreactivity for fibronectin (**D**) and CD49c (**E**). In passage-4 RPE cells, CRALBP (**G**) and RPE65 (**H**) do not show immunoreactivity, other than fibronectin (**I**) and CD49c (**J**), which is found positive in passage-4 cells. Nuclei were stained with 4′6-diamidino-2-phenylindole (DAPI). **A** and **F** show Nomarski imaging of the respective RPE cells, native (**A**) and passage-4 (**F**).

Interestingly, 28 proteins were only expressed in native RPE cells (25% of all 112 identified proteins), (Table 1; proteins 24–51) and they were not detectable in cultured RPE cells (Table 1). Among these were cellular retinaldehyde-binding protein (CRALBP) (Table 1, protein 29), retinol dehydrogenase 5 (RDH5) (Table 1, protein 37) and retinal pigment epithelium-specific protein 65 kDa (RPE65) (Table 1, protein 38), all proteins known to be expressed in RPE cells [15,16]. RPE65 is a RPE cell specific protein, which is only expressed in original RPE cells [17]. In contrast, 61 proteins were exclusively expressed in RPE cells of passage-4 (54.5% of all 112 identified proteins), for example CD49c, fibronectin and thrombospondin 1 (Table 1, proteins 70, 85 and 108). We picked CRALBP, RPE65, fibronectin and CD49c and validated these differentially regulated proteins on transcriptomic level by PCR (Figure 2), on protein level by Western blot (Figure 3) and by immunocytochemistry (Figure 4). According to the results from LC MS/MS, CRALBP and RPE65 were expressed in native RPE cells but not in passage-4 cells (Figures 2–4).

By immunocytochemistry, a cytoplasmatic punctuate expression pattern could be shown for CRALBP, whereas RPE65 showed positive immunoreactivity throughout the cytoplasm and parts of the membrane in native RPE cells. Fibronectin and CD49c were present in passage-4 RPE cells and absent in native cells, which could be shown by immunoblotting and immunocytochemistry (Figures 3 and 4). Immunocytochemistry of passage-4 cells showed a perinuclear staining for fibronectin and a punctuate staining for CD49c. On mRNA level, a distinct signal in passage-4 RPE cells and only a faint signal in native cells for fibronectin and CD49c could be demonstrated. Beta-actin was abundant in both states, native and passage-4 (Figures 2 and 3).

## 3. Discussion

The RPE forms the outer blood-retinal barrier and plays an essential role in visual function [18]. Since it is located between the choroids and the neurosensory retina, it has to fulfill important functions, like absorption of light, reisomerization of all-trans retinal into 11-cis retinal, protection against photooxidation, epithelial transport of ions, nutrients and fluids, phagocytosis of photoreceptor outer segments, secretion of essential factors for the integrity of neighboring tissues and supporting the immune privilege of the inner eye [19]. As one characteristic of ERU is the breakdown of the outer blood-retinal barrier, our aim is to elucidate the pathomechanisms that are involved in this breakdown. Therefore, performing functional studies on equine RPE cells will be necessary to understand their role in ERU [20]. We set our focus on cell surface membrane proteins in this study, as we expect them to be targets for initiating events in the breakdown of the outer blood-retinal barrier in eyes of ERU diseased horses. In order to detect differentially regulated cell surface membrane proteins in the eyes of affected horses, it is a prerequisite to identify the cell surface membrane proteome of equine RPE cells in the physiological state. We compared native equine RPE cells with passage-4 cells to determine if there were any changes in this proteome due to cultivation and dedifferentiation. Changes in the whole proteome without specific focus on cell surface membrane proteins in cultured human RPE cells compared to native human RPE cells were described previously [14]. To our knowledge, we are the first to describe the cell surface membrane proteome of native and cultured equine RPE.

A total of 112 proteins were identified by LC MS/MS, of which 84% were cell membrane proteins (Table 1). Twenty-three proteins were concurrently expressed in both, native and passage-4 RPE cells which equals 20.5% of all 112 identified proteins (Table 1, proteins 1–23). Whereas 28 proteins (25% of all 112 identified proteins) were exclusively expressed in native cells (Table 1, proteins 24–51), 61 proteins (54.5% of all 112 identified proteins) were expressed only in passage-4 cells (Table 1, proteins 52–112), indicating a profound re-organization of the cell surface proteome in cultured equine RPE cells.

As expected, beta-actin, an ubiquitously expressed housekeeping protein [21], was equally expressed in both examined states (Table 1, protein 3). Among the equally expressed proteins, six cytokeratins were present, which are intermediate filament proteins (Table 1, proteins 7–12). Desmoplakin, a desmosomal protein which mediates intercellular adhesion in human RPE cells [22], was also equally expressed in both states of equine RPE cells (Table 1, protein 13), as well as basigin (Table 1, protein 2), which plays a key role in extracellular matrix (ECM), remodeling [23] and neuroplastin (Table 1, protein 18), which belongs to the same protein family [24]. Basigin was already described as being expressed by human RPE *in situ* and by a human RPE cell line, namely ARPE-19 [25]. Further, four different types of Na^+^/K^+^ ATPases were identified, which are usually localized apically in RPE cells [26]. Three of them were expressed in both states (Table 1, proteins 15–17), whereas one of them was only present in passage-4 RPE cells (Table 1, protein 98).

Among the 28 proteins exclusively expressed in native equine RPE cells, there were three cytokeratins (Table 1, proteins 31–33) and few ion-channel proteins, namely Kir7.1; solute carrier family 1, member 4; solute carrier family 12, member 2; solute carrier family 13, member 3; solute carrier family 16, member 1; solute carrier family 4, member 7; solute carrier family 6, members 6, 9, 13, 20; and solute carrier organic anion transporter family, member 1A2 and member 1B3 (Table 1, proteins 39–49). Further, we identified two RPE cell markers, CRALBP and RPE65 [16] (Table 1, proteins 29 and 38). CRALBP, RPE65 and also RDH5, which was exclusively expressed by native RPE cells (Table 1, protein 37), are proteins playing a key role in the visual cycle [27–29]. CRALBP, RPE65 and RDH5 were abundant in native and absent in cultured RPE cells. Correlating with our results, former research observed that RPE65 and CRALBP, both proteins associated with highly specialized functions of the RPE, were absent in cultured RPE cells [14,30]. Regarding that CRALBP is not only expressed by RPE cells, but also, for instance, by Mueller glial cells [31], we believe that RPE65 is an excellent marker for the verification of native in comparison to cultured RPE, as it was found to be differentially expressed by Western blot analysis, immunocytochemistry and even PCR (Figure 2–4). Another reason for considering RPE65 an attractive protein for further research in ERU is the fact that immunoreactions against RPE65, next to CRALBP and S-Antigen, are known to induce experimental autoimmune uveitis in rats [7,32,33].

Besides cell surface proteins, which were only expressed by native equine RPE cells (Table 1, proteins 23–51), there were 61 proteins exclusively detected in cultured passage-4 RPE cells (Table 1, proteins 52–112). Interestingly, there was a notable increased expression of several clusters of differentiation (CD) molecules in passage-4 cells (Table 1, proteins 59–79) compared to the native state, whereas ion channel protein expression decreased (Table 1). An altered expression of ECM proteins could be observed as well, for instance of fibulin 1, fibulin 2, fibronectin and thrombospondin 1. (Table 1, proteins 85–87 and 108). Thrombospondin 1 is not only an ECM protein expressed by RPE cells, but it is also known to suppress activated T-cells [34], which additionally makes this protein very interesting for ERU research. It was found to be exclusively expressed in cultured equine RPE cells. Fibronectin was also absent in native equine RPE cells, but abundant in passage-4 cells (Table 1, protein 85; Figures 3 and 4). Immunocytochemically, fibronectin shows positive immunoreactivity with a perinuclear expression pattern in passage-4 cells, and no immunoreactivity was observed in native RPE cells (Figure 4). *In vivo*, the RPE basement membrane contains ECM proteins such as laminin, fibronectin, vitronectin and collagen type VI [35]. When RPE is dissociated from the eyecup, the RPE basement membrane remains in the eyecup, and there is no matrix for the RPE cells to attach to *in vitro*. In order to adhere and form a monolayer of epithelial cells *in vitro*, RPE cells begin to produce ECM proteins, such as fibronectin, themselves [36,37]. The perinuclear expression pattern in passage-4 indicates an augmented protein synthesis of the ECM protein fibronectin in passage-4 cells. Furthermore, there was an altered expression of proteins associated with cell adhesion, predominantly integrins (Table 1, proteins 69–72, 76 and 92–95), that are known to not only interact with ECM proteins [38], but also cadherins, catenin, cell adhesion molecule 1, dystroglycan 1, vitronectin and versican (Table 1, proteins 55, 56, 58, 73, 80, 81 and 112). CD49c, which is also known as integrin-alpha 3, belongs to a family of receptors for ECM and cell adhesion molecules [39]. Gullapalli *et al.* observed an increased expression of CD49c on transcriptomic and proteomic level in cultured human RPE cells [40], whereas uncultured human RPE cells did not express or only expressed low amounts of CD49c. These findings were consistent with their real-time PCR results [40]. We were able to demonstrate an altered expression in passage-4 cells as well, compared to native cells on mRNA and proteomic level (Table 1, protein 70; Figures 2–4). In Western blot analysis, no signal in the lane with native RPE cell lysate was detectable, whereas the lane with passage-4 cell lysate showed a distinct signal. Also, no staining for CD49c could be shown in native cells by immunocytochemistry, confirming CD49c expression on proteomic level to be specific for passaged RPE cells (Figures 2–4).

Interestingly, we showed that the cell surface proteome of equine RPE cells shifted from proteins with RPE specialized functions (CRALBP, RPE65 and RDH5) in native cells (Table 1, proteins 29, 37 and 38; Figures 2–4) to proteins that are associated with cell adhesion and ECM formation (integrins, cell adhesion molecule 1, catenin, thrombospondin 1, fibronectin) in cultured cells (Table 1, proteins 58, 80, 85, 108 and 93–95; Figures 2–4).

The altered expression of molecules that are associated with cell adhesion in passage-4 cells could be clearly demonstrated in Figure 1 (dark green segments). According to Figure 1A, in native equine RPE cells, only 2.29% of all respective proteins are associated with cell adhesion (dark green segment), whereas in passage-4 cells, 12.06% of the expressed proteins are capable of cell adhesion as their biological process (Figure 1B, dark green segment). These observations in equine cells correspond to the findings in cultured human cells where a shift to processes such as cell adhesion could be accordingly displayed [14]. Another notable increase in passage-4 RPE cells could be seen in cell communication (Figure 1A,B, grey segments). As the normal function of the RPE depends on the maintenance of tight adhesions between the cells [41], and cell-cell adhesion and intercellular communication are integrated [42], it is not surprising that this biological process alters concurrently in passage-4 RPE cells (Figure 1A,B, grey segments). Interestingly the relation between cell adhesion and communication remains equal in both states (Figure 1A,B, grey segments).

In contrast to alteration of proteins associated with cell adhesion and communication in passage-4 cells, Figure 1 demonstrates a noticeable decrease of proteins that are associated with transport as their biological process in passage-4 cells (6.74%) (Figure 1B, bright blue segment) compared to the native state (34.86%) (Figure 1A, bright blue segment), correlating with the observations in Table 1, where in passage-4 cells a strong decrease in ion transporting channels was noted.

Our results therefore clearly demonstrate that there is a profound re-organization of the cell surface membrane proteome in cultured equine RPE cells compared to native RPE cells. We believe that one reason for these differences is most probably the *in vitro* situation, which profoundly differs from the situation *in vivo*, where RPE is provided with environmental specific factors by surrounding tissue that are not included in standard cell culture media [43]. The lack of the choroid on the basal side and the retina on the apical side of the cells in culture could lead to a decrease of functions like transport, which is not needed in its entirety *in vitro*. Then again, other functions, as cell-adhesion, become more important. Since retinal tissue is destroyed and retinal architecture is widely disintegrated in ERU [13], we assume that the proteomic changes in diseased eyes are very similar to the changes observed in this study, as disintegrated and destroyed retinae could be compared to a missing retina in culture. In some ways, culture is like a disease state, where a degenerate neural retina no longer sends RPE the signals that modulate key functions [11]. Another reason for the differences in cell surface proteomes of native and cultured RPE cells is that RPE cells do not divide *in vivo*, in contrast to RPE cells *in vitro*[44]. All of this may lead to an altered expression of proteins, as the proteome is dynamic and changes with the physiological state of the cell [14]. In the past, changes in protein expression due to *in vitro* culturing and passaging, could be observed in cells other than RPE cells as well [45,46]. Our results clearly validated this and demonstrated that, by culturing equine RPE cells, changes in the cell surface proteome occur and that the passage-4 cells only to a limited extend show properties of native physiological RPE cells.

Regarding the short time post mortem until further processing of the equine eyes used in this study in comparison to eyes obtained from human donors, we were able to perform analyses on native RPE cells without loss of proteins due to degradation. By means of biotinylation and LC MS/MS in order to capture, enrich and identify cell surface membrane proteins, we were the first to characterize the cell surface proteome of RPE cells of two different states, native and passage-4. Detecting a total of 28 CD molecules, we contributed to a profile and repertoire of RPE CD antigens of which, to our knowledge, ten have not yet been described to be expressed in RPE cells of any species before (Table 1, proteins 25–27, 59, 60 and 64–67). We believe that a cell surface proteome of healthy equine RPE cells is essential and a necessity in order to compare RPE cells of healthy physiological and diseased pathological state in the future, and thus provides the basis for further research in this field. Furthermore, knowledge of the cell surface proteome of equine RPE cells is not only of great value for research of ERU, but also of major importance for research in the human field of other severe eye diseases with participation of the RPE as ocular tissue, namely Age Related Macular Degeneration (AMD), human autoimmune uveitis and Proliferative Vitreoretinopathy (PVR) [47–49]. Since ERU is the only spontaneous model for human autoimmune uveitis [50], it is of great importance to investigate and understand the pathomechanisms leading to this severe disease. The lack of proteins with highly specialized RPE functions in cultured RPE compared to native cells requires the use of native cells in research. Therefore, we believe that primary cells should be used for experiments in which it is important to evaluate changes in these specialized proteins in healthy versus diseased RPE cells.

Since cultured cells show a great tendency to form intercellular adhesions, these cells could be used for functional studies where tight junctional formation is required, for example as transmigration assays on semi-permeable filter systems. In regard to availability, cultured cells have a significant advantage for the use in research compared to native cells. Further, they can be passaged, and thus multiplied, enabling functional studies to be performed with a much higher amount of cells and a higher diversity of experimental setups.

Nevertheless, future proteomic studies on diseased equine RPE cells have to be conducted to clarify if the shift of the cell surface proteome in cultured equine RPE cells to proteins, which were not detectable in native cells, could be similar to equine RPE cells in diseased state, as was already shown for protein expression profile of dedifferentiated human RPE cells and initial stages of PVR [14].

To summarize, we were the first to provide a characterization of the cell surface proteome of native and cultured equine RPE cells by means of cell surface protein biotinylation and high resolution mass spectrometry. Additionally, we showed that there is a considerable difference between native and cultured equine RPE cells regarding their cell surface protein expression by investigation of differentially expressed proteins. Additionally, we were the first to describe ten CD molecules in equine RPE cells, which, to our knowledge, have not yet been described in the context of RPE cells of any species (Table 1, proteins 25–27, 59, 60 and 64–67). Cultured RPE cells experience a profound re-organization of the cell surface proteome since only 20.5% of all 112 identified proteins were expressed equally in both cell states. An interesting question that arises is how quickly the proteome of equine RPE cells changes after preparation of native cells and subsequent cultivating. As we set our focus on the comparison between native RPE cells and cells of passage-4, future experiments need to be conducted to evaluate if and when this proteomic shift or a tendency is notable in passage-2 and -3 cells. Nevertheless we believe that we have set a great foundation with regard to future experimental studies on the function of RPE cells in physiological and pathological conditions.

## 4. Experimental Section

### 4.1. Preparation of Equine Retinal Pigment Epithelium Cells

No experimental animals were used in this study. Eyes providing healthy equine retinal pigment epithelium samples were obtained at a local abattoir. Eyes were considered normal based on the diagnosis of the veterinarian present at the abattoir, medical histories of the horses as provided by their owners and preliminary histological analysis. The collection and use of equine eyes from animals that were killed due to a research-unrelated cause, was approved for purposes of scientific research by the appropriate board of the veterinary inspection office Munich, Germany (Permit number: 8.175.10024.1319.3). RPE cells from eyes of eight different eye-healthy horses were used in this study, four biological replicates of native cells and four biological replicates of passage-4 cells. For each verification procedure, we had at least two technical replicates (two for PCR and immunocytology, four in Western blots).

RPE cells were prepared as follows: First, residual periocular tissue was carefully removed with forceps and scissors. Then, the intact eyes were rinsed in 70% Ethanol for 2 min followed by a short washing step in cold phosphate buffered saline (PBS). Afterwards, eyes were stored in sterile Dulbecco’s Modified Eagle Medium (DMEM) until further processing. All following preparation steps were performed under a laminar flow hood with sterile instruments. Eye globes were cut open circumferentially, and anterior parts of the eye, vitreous and neurosensory retina, were carefully removed. Posterior eyecups were rinsed twice with 37 °C pre-warmed PBS to wash clear of residual vitreous and retinal tissue without destroying the RPE layer. For complete removal of retinal tissue, especially the rod outer segments, eyecups were filled with pre-warmed 1mM PBS/EDTA pH 7.4 and incubated at room temperature (RT) for 15 min. The PBS/EDTA solution was discarded and, in order to enzymatically detach the RPE cell layer, eyecups were filled with pre-warmed dissociation buffer containing 1 μL papain (CellSystems, Troisdorf, Germany) per milliliter buffer (1 mM PBS/EDTA pH 7.4, 260 mM l-Cysteine, Sigma Aldrich, Deisenhofen, Germany, 1% BSA, AppliChem, Darmstadt, Germany). The eyecups were incubated at 37 °C and 5% CO^2^ atmosphere for 25 min. Then, the RPE cells were resuspended in dissociation buffer within the eyecup. The enzymatic activity of papain was blocked by transferring the suspension containing the RPE cells into DMEM supplemented with 10% heat-inactivated fetal calf serum (FCS) and 1% penicilline/streptomycine (P/S). The cell suspension was centrifuged at RT, 130× *g* for 5 min, and the resulting cell pellet was carefully washed twice with pre-warmed PBS. Finally, the RPE cells were either seeded into T25 cell culture flasks (Sarstedt, Nümbrecht, Germany), or biotinylated and processed for mass spectrometry. For passage-4 cells, RPE cells were cultured in DMEM supplemented with 10% heat-inactivated FCS and 1% P/S. The cells were maintained at 37 °C and 5% CO^2^ atmosphere until they reached confluence after 14 days of cultivation. For protein expression analysis by mass spectrometry, cells were harvested with Trypsin/EDTA (Biochrom, Berlin, Germany), washed twice with cold PBS and centrifuged in between washing steps at 4 °C, 500× *g* for 10 min. Prior to cell lysis, cell surface membrane proteins of 1.6 × 10^6^ passage-4 RPE cells and 5.0 × 10^5^ native RPE cells were labeled with 542 μg biotin and 50 μg biotin, respectively. After 30 min rotation at 4 °C and two centrifugation steps at 2800× *g* and 16,000× *g* for 10 min each at 4 °C, cells were lysed with 1% Nonidet P-40, 150 mM NaCl, 1 × Roche Complete Protease Inhibitor, EDTA-free; 5 mM 2-Iodacetamide in 10 mM Tris-HCl pH 7.6 and biotinylated cell surface proteins were captured using Streptavidin-beads (IBA, Goettingen, Germany). After extensive washing to remove non-specifically bound proteins, captured proteins were cleaved through digesting beads overnight with trypsin (Promega, Mannheim, Germany) followed by incubation with glycerol-free PNGase F (New England Biolabs, Frankfurt/Main, Germany) at 37 °C. Finally, peptides were analyzed by LC-MS/MS.

### 4.2. Mass Spectrometry

LC-MS/MS mass spectrometry was performed as previously described [1,5]. Briefly, cell lysates were subjected to on-membrane trypsin digest. Resulting peptides were separated on a reversed phase chromatography column (PepMap, 15 cm × 75 μm ID, 3 μm/100A pore size, LC Packings), which was operated on a nano-HPLC apparatus (Ultimate 3000, Dionex, Idstein, Germany) connected to a linear quadrupole ion-trap Orbitrap (LTQ Orbitrap XL, Thermo Fisher Scientific, Schwerte, Germany). The mass spectrometer was operated in the data-dependent mode to automatically switch between Orbitrap-MS and LTQ-MS/MS acquisition. Survey full scan MS spectra (from *m*/*z* 300 to 1500) were acquired in the Orbitrap resolution *R* = 60,000 at *m*/*z* 400. The method used allowed parallel selection of up to the ten most intense ions for fragmentation on the linear ion trap using collision induced dissociation at a target value of 100,000 ions and subsequent dynamic exclusion for 30 s. MS/MS spectra were exported from the Progenesis software as Mascot Generic file (mgf) and used for peptide identification with Mascot (version 2.3.02, Matrix Science: London, UK; available online: http://www.matrixscience.com, accessed on 26 June 2012) in the Ensembl database for horse (Equus caballus; EquCab2.56.pep, available online: ftp://ftp.ensembl.org/pub/current_fasta/equus_caballus/pep/, accessed on 26 June 2012) containing a total of 22641 protein sequences. A protein was considered as identified if the confidence score was higher than 30 at a significance threshold for the Mascot result of *p* ≤ 0.01.

### 4.3. Gene Ontology (GO)

Gene Ontology annotations were retrieved from Ensembl and EMBL-EBI QuickGO database. GO annotations were available for all identified proteins and were classified based on three organizing principles of GO, namely: biological process, cellular component and molecular function. GO annotations for each protein were hierarchically summarized using AmiGO. Based on these summarized GO annotations pie charts were made, illustrating differences in biological processes, cellular component and molecular function between native and passage-4 equine RPE cells. In this manuscript pie charts representing differences in biological processes of native and passage-4 RPE cells are presented. Biological processes, which are represented by 1.1% or less of all proteins, are summarized in the field “others” (Figure 1A,B).

### 4.4. Polymerase Chain Reaction (PCR)

RNA was isolated from freshly isolated equine RPE and cultured RPE of passage-4 using RNeasy Mini Kit (Qiagen, Hilden, Germany) according to the manufacturer’s protocol. Using RevertAid First Strand cDNA Synthesis Kit (Thermo Scientific, Schwerte, Germany), 2 μg of RNA were reverse transcribed in presence and absence of reverse transcriptase, according to the manufacturer’s protocol. PCR was then performed in a total volume of 50 μL using the cDNA as a template in the presence of specific primers for the respective proteins. Reaction buffer, dNTPs and Taq DNA Polymerase were obtained from Fermentas (St. Leon-Rot, Germany). The thermocycler, which was used for PCR, was a Tetrad PTC-225 Thermal Cycler (MJ research, St. Bruno (Quebec), Canada). PCR, which was performed on samples that were reverse transcribed to cDNA in the absence of reverse transcriptase, did not show any amplified product. Amplified products were separated by 2% agarose gel electrophoresis, stained with Serva DNA Stain G (Serva Electrophoresis GmbH, Heidelberg, Germany) and visualized with UV illumination (Herolab UVT-40M, Wiesloch, Germany) (Figure 2).

### 4.5. Western Blot Analysis

Retinal pigment epithelial cell pellets were solubilized in lysis buffer (9 M urea, 2 M thiourea, 1% DTT, 4% CHAPS, and 2.5 mM each of EGTA and EDTA), and protein content was quantified with Bradford assay (Sigma-Aldrich, Deisenhofen, Germany). Equal total protein amounts (5 μg) of RPE samples were resolved in 1D SDS gels and then blotted semidry onto polyvinyl-difluoride membranes (GE Healthcare, Freiburg, Germany). Unspecific binding was blocked with 5% non-fat dry milk in PBS with 0.05% Tween20 (PBS-T) and 5% goat serum. Blots were incubated with primary antibodies overnight at 4 °C. For verification, we used the following primary antibodies: mouse anti-human beta-actin (Sigma-Aldrich, Deisenhofen, Germany; dilution: 1:7000), rabbit anti-human RPE65 (Santa Cruz, Heidelberg, Germany; dilution: 1:800), anti-equine CRALBP antiserum (self-made; dilution: 1:1000) [7]), rat anti-human CD49c (Ascenion, München, Germany; reported cross-reactivity to horse, dilution: 1:500) and rabbit anti-human Fibronectin (Thermo Scientific, Schwerte, Germany; dilution: 1:1000). After washing, blots were incubated with respective peroxidase-coupled (POD) secondary antibodies (goat anti-rat IgG POD, Sigma-Aldrich, Deisenhofen, Germany; dilution: 1:3000; goat anti-rabbit IgG POD, Sigma-Aldrich, Deisenhofen, Germany; dilution: 1:3000; goat anti-mouse IgG POD, Sigma-Aldrich, Deisenhofen, Germany, dilution: 1:5000; and goat anti-horse IgG Fc-POD, Biozol, Eching, Germany; dilution: 1:10000). Signals were detected by enhanced chemoluminescence (ECL) on X-ray films (Christiansen, Planegg, Germany). Protein expression was quantified after imaging the signals on a transmission scanner using LabScan 5.0 software (GE Healthcare, Freiburg, Germany, 2003) and densitometric analysis with ImageQuantTL software (GE Healthcare, Freiburg, Germany, 2003). Signals were normalized to beta-actin content after staining of lanes with monoclonal mouse anti-beta-actin antibody (Sigma-Aldrich, Deisenhofen, Germany; dilution: 1:7000) prior to statistical analysis. As Kolmogorov-Smirnov test showed that data were not distributed normally (*p* ≤ 0.05). Mann-Whitney test was applied for calculation of statistical significance (*p* ≤ 0.05). Statistical analysis was performed using Paleontological Statistics software (PAST, available online: http://folk.uio.no/ohammer/past/index.html, accessed on 3 July 2012).

### 4.6. Immunocytochemistry

For immunocytochemistry, native equine RPE cells were set onto glass slides immediately after obtaining these cells from equine eye globes. Equine RPE cells of passage-4 were trypsinized in order to detach them from the cell culture flask and then set onto glass slides. Glass slides were then fixed in ice cold acetone for 10 min, followed by blocking with 1% BSA in PBS-T and 5% normal goat serum for 40 min at RT to prevent unspecific antibody binding. For fluorescence labeling, slides were incubated overnight at 4 °C with the following primary antibodies: mouse anti-human CRALBP (Santa Cruz, Heidelberg, Germany; dilution: 1:50), rabbit anti-human RPE65 (Santa Cruz, Heidelberg, Germany; dilution: 1:200), rabbit anti-human Fibronectin (Thermo Scientific, Schwerte, Germany; dilution: 1:500) and rat anti-human CD49c (Ascenion, München, Germany; reported cross-reactivity to horse; dilution: 1:200). Glass slides were then incubated with secondary antibodies for 30 min at RT (Alexa Fluor 546 goat anti-mouse, Alexa Fluor 546 goat anti-rabbit, Alexa Fluor 546 goat anti-rat; Invitrogen, Karlsruhe, Germany; dilution: 1:500). Cell nuclei were counter-stained with 4′,6 Diamidino-2-phenylindole (DAPI; Invitrogen, Karlsruhe, Germany; dilution: 1:1000). Finally, glass slides were mounted with glass cover slips using Dako fluorescent mounting medium (Dako, Hamburg, Germany). Fluorescent images were recorded using Axio Imager M1 or Z1 (Zeiss, Göttingen, Germany) and Axio Vision 4.6 software (Zeiss, Göttingen, Germany).

## 5. Conclusions

The results of this study indicate a profound re-organization of the cell surface proteome of passage-4 equine RPE cells compared to native cells. We provided the first proteome profile of cell surface proteins of native and cultured equine RPE cells by means of biotinylation and LC MS/MS. We believe that our findings are a prerequisite and of great value, not only in future ERU research, but also in research of other ocular diseases with participation of the RPE, for example AMD, human autoimmune uveitis or PVR. In our future studies, we will consider the differences in protein expression of native and cultured RPE cells, thus enhancing the transferability of *in vitro* studies to the actual pathomechanisms *in vivo*.

## Figures and Tables

**Table 1:** Cell surface proteins expressed by RPE cells of horses. Proteins listed were identified by LC MS/MS with a probability score that is significant with *p* < 0.05 if the confidence score was >30 at a significance threshold for the Mascot result of *p* ≤ 0.01. N = native RPE cells; P4 = passage-4 RPE cells; C = cytoplasmatic; M = membrane associated; E = extracellular.

#	Identified protein	Accession number	MW (kDa)	Occurrence		Unique peptide count		Sequence coverage (%)		Cellular localization		
			
				N	P4	N	P4	N	P4	C	M	E
1	ATP-binding cassette, sub-family C, member 1	ENSECAP00000005390	164	x	x	9	17	9	17		x	
2	Basigin	ENSECAP00000010130	24	x	x	4	3	28	28		x	
3	Beta-actin	ENSECAP00000013637	41	x	x	2	3	8	23	x		
4	CD29	ENSECAP00000020201	88	x	x	6	21	11	34		x	
5	CD44	ENSECAP00000008636	77	x	x	2	7	4	14	x	x	
6	CD90	ENSECAP00000007841	18	x	x	3	4	24	30		x	
7	Cytokeratin 1	ENSECAP00000019935	66	x	x	3	3	6	7	x	x	
8	Cytokeratin 2	ENSECAP00000015380	61	x	x	3	3	9	9	x		
9	Cytokeratin 16	ENSECAP00000004544	51	x	x	6	4	19	13	x		
10	Cytokeratin 10	ENSECAP00000018005	43	x	x	14	14	28	30	x		
11	Cytokeratin 5	ENSECAP00000018010	62	x	x	11	8	17	14	x	x	
12	Cytokeratin 6C	ENSECAP00000011363	60	x	x	2	3	12	13	x		
13	Desmoplakin	ENSECAP00000012688	168	x	x	4	4	4	5		x	
14	Ectonucleotide pyrophosphatase/phosphodiesterase 1	ENSECAP00000011146	96	x	x	5	15	10	28		x	x
15	Na(+)/K(+) ATPase alpha-1 subunit	ENSECAP00000022397	113	x	x	2	3	13	12		x	
16	Na(+)/K(+) ATPase alpha-3 subunit	ENSECAP00000022127	113	x	x	10	5	15	8		x	
17	Na(+)/K(+) ATPase beta-1 subunit	ENSECAP00000015566	35	x	x	3	3	14	14		x	
18	Neuroplastin	ENSECAP00000006428	42	x	x	2	5	6	16		x	
19	Plexin B2	ENSECAP00000017324	183	x	x	3	10	3	11	x	x	
20	Pyruvate Carboxylase	ENSECAP00000022492	130	x	x	5	13	7	18		x	
21	Solute carrier family 2, member 1	ENSECAP00000000404	54	x	x	7	5	12	9	x	x	
22	Solute carrier family 3, member 2	ENSECAP00000007939	62	x	x	10	10	28	30	x	x	
23	Solute carrier family 44, member 2	ENSECAP00000009162	79	x	x	2	3	3	8		x	
24	Carbonic anhydrase 14	ENSECAP00000002354	31	x		4	0	25	0		x	
25	CD107a	ENSECAP00000015399	44	x		2	1	8	4		x	
26	CD107b	ENSECAP00000016219	46	x		2	1	5	2		x	
27	CD156c	ENSECAP00000006414	82	x		2	0	6	0	x	x	
28	CD36L2	ENSECAP00000022283	50	x		2	0	7	0		x	
29	Cellular retinaldehyde-binding protein	ENSECAP00000002273	36	x		3	0	12	0	x		
30	Chondroitin sulfate proteoglycan 5	ENSECAP00000020923	54	x		2	0	9	0		x	
31	Cytokeratin 17	ENSECAP00000014186	48	x		3	1	14	7	x	x	
32	Cytokeratin 75	ENSECAP00000008235	59	x		2	0	12	7	x		
33	Cytokeratin 77	ENSECAP00000006788	63	x		2	1	8	5	x		
34	Glycoprotein M6A	ENSECAP00000014508	31	x		5	0	18	0		x	
35	Heat shock protein 90 kDa beta member 1	ENSECAP00000009012	92	x		2	1	4	2	x	x	
36	Inward rectifier K(+) channel Kir7.1	ENSECAP00000005853	41	x		3	0	9	0		x	
37	Retinol dehydrogenase 5	ENSECAP00000017236	35	x		3	0	15	0		x	
38	RPE65	ENSECAP00000008347	60	x		3	0	11	0		x	
39	Solute carrier family 1, member 4	ENSECAP00000009506	55	x		3	1	8	4	x	x	
40	Solute carrier family 12, member 2	ENSECAP00000013091	119	x		4	1	5	2	x	x	
41	Solute carrier family 13, member 3	ENSECAP00000008631	63	x		3	0	7	0		x	
42	Solute carrier family 16, member 1	ENSECAP00000019149	54	x		3	0	7	0		x	
43	Solute carrier family 4, member 7	ENSECAP00000004107	137	x		2	0	3	0		x	
44	Solute carrier family 6, member 13	ENSECAP00000012525	68	x		4	0	9	0		x	
45	Solute carrier family 6, member 9	ENSECAP00000003659	71	x		3	0	7	0		x	
46	Solute carrier family 6, member 6	ENSECAP00000011341	70	x		2	0	5	0		x	
47	Solute carrier family 6, member 20	ENSECAP00000018972	66	x		3	0	7	0		x	
48	Solute carrier organic anion transporter family, member 1A2	ENSECAP00000015472	74	x		2	0	3	0		x	
49	Solute carrier organic anion transporter family, member 1B3	ENSECAP00000010749	76	x		4	0	9	0		x	
50	Thioredoxin domain containing 5	ENSECAP00000000114	36	x		2	0	12	0		x	
51	Transmembrane protein 27	ENSECAP00000017054	25	x		3	0	18	0		x	
52	Actin, alpha 1	ENSECAP00000000126	42		x	0	5	0	18	x		
53	Adlican	ENSECAP00000015067	313		x	0	5	0	3			x
54	Anoctamin 6	ENSECAP00000010736	105		x	0	3	0	7		x	
55	Cadherin 13	ENSECAP00000018588	76		x	0	7	0	15		x	
56	Cadherin 2	ENSECAP00000008264	91		x	0	8	0	21		x	
57	Calcium channel, voltage-dependent, alpha 2/delta subunit 1	ENSECAP00000008740	104		x	0	5	0	8		x	
58	Catenin, gamma	ENSECAP00000018374	82		x	1	2	2	3	x	x	
59	CD105	ENSECAP00000018048	70		x	0	4	0	11		x	x
60	CD109	ENSECAP00000020689	159		x	0	7	0	9			x
61	CD13	ENSECAP00000007954	110		x	0	2	0	3		x	
62	CD140b	ENSECAP00000022400	121		x	1	11	2	17		x	
63	CD142	ENSECAP00000020955	32		x	1	2	6	10		x	x
64	CD280	ENSECAP00000010799	162		x	0	9	0	9		x	
65	CD315	ENSECAP00000014826	98		x	1	3	2	5		x	
66	CD318	ENSECAP00000022716	93		x	1	2	2	4		x	
67	CD362	ENSECAP00000006433	22		x	0	2	0	12		x	
68	CD46	ENSECAP00000007802	32		x	0	2	0	10		x	
69	CD49a	ENSECAP00000015387	129		x	0	9	0	13		x	
70	CD49c	ENSECAP00000013928	119		x	1	9	1	12		x	
71	CD49d	ENSECAP00000009001	114		x	0	5	0	7		x	
72	CD49e	ENSECAP00000022617	115		x	0	7	0	11		x	
73	CD51	ENSECAP00000020829	113		x	0	15	0	21		x	
74	CD54	ENSECAP00000011996	57		x	0	2	0	0		x	x
75	CD56	ENSECAP00000017648	93		x	0	3	0	6		x	
76	CD61	ENSECAP00000016485	84		x	0	3	0	8		x	
77	CD71	ENSECAP00000021947	86		x	0	2	0	4		x	x
78	CD73	ENSECAP00000007576	51		x	0	8	0	28	x	x	
79	CD91	ENSECAP00000010929	504		x	0	44	0	16		x	
80	Cell adhesion molecule 1	ENSECAP00000013033	47		x	0	2	0	12		x	
81	Dystroglycan 1	ENSECAP00000007588	97		x	0	3	0	6	x	x	
82	Endothelin converting enzyme 1	ENSECAP00000014392	86		x	0	2	0	5	x	x	
83	EPH receptor A2	ENSECAP00000006952	108		x	0	4	0	5		x	
84	Fibroblast activation protein, alpha	ENSECAP00000010111	88		x	0	4	0	8		x	
85	Fibronectin 1	ENSECAP00000005228	262		x	0	35	0	26		x	x
86	Fibulin 1	ENSECAP00000016104	75		x	0	6	0	16		x	X
87	Fibulin 2	ENSECAP00000007213	125		x	1	17	3	24			x
88	Folate hydrolase 1	ENSECAP00000020544	85		x	0	2	0	5	x	x	
89	Frizzled family receptor 1	ENSECAP00000000907	60		x	0	2	0	4		x	
90	Frizzled family receptor 2	ENSECAP00000015209	61		x	0	2	0	4	x	x	
91	Immunoglobulin superfamily containing leucine-rich repeat	ENSECAP00000002466	46		x	0	3	0	11			x
92	Integrin alpha FG-GAP repeat containing 3	ENSECAP00000022543	60		x	0	5	0	14		x	
93	Integrin, alpha 11	ENSECAP00000020852	133		x	0	7	0	12		x	
94	Integrin, alpha 8	ENSECAP00000011756	117		x	0	8	0	16		x	
95	Integrin, beta 5	ENSECAP00000006461	85		x	1	11	3	22		x	
96	Latent transforming growth factor beta binding protein 2	ENSECAP00000016809	196		x	0	2	0	2			x
97	MHC I	ENSECAP00000008517	40		x	0	2	0	7		x	
98	Na, K-ATPase beta-3 polypeptide	ENSECAP00000017575	27		x	0	2	0	15	x	x	
99	Natriuretic peptide receptor C	ENSECAP00000017782	60		x	0	4	0	12		x	
100	Neuropilin 1	ENSECAP00000018748	103		x	0	8	0	15	x	x	
101	Neuropilin 2	ENSECAP00000015439	105		x	0	2	0	4		x	
102	NOTCH2	ENSECAP00000013532	245		x	0	2	0	2		x	
103	Plexin domain containing 2	ENSECAP00000015262	44		x	0	4	0	13		x	
104	Propionyl CoA carboxylase, alpha polypeptide	ENSECAP00000016512	56		x	0	4	0	13	x		
105	Protein tyrosine kinase 7	ENSECAP00000009553	98		x	0	11	0	21		x	
106	Solute carrier family 1, member 5	ENSECAP00000009592	56		x	0	3	0	6		x	
107	Sushi domain containing 5	ENSECAP00000005951	67		x	0	4	0	11		x	
108	Thrombospondin 1	ENSECAP00000007423	130		x	0	27	0	37		x	x
109	Transmembrane protein 2	ENSECAP00000019147	154		x	0	3	0	3		x	
110	Tubulin, alpha 1c	ENSECAP00000007419	50		x	0	2	0	7	x		
111	Vasorin	ENSECAP00000002864	72		x	0	3	0	5		x	
112	Versican	ENSECAP00000017347	94		x	0	2	0	4			x
